# Introspection of UBNIN and Modified-UBNIN Algorithms for Structural MRI. Reply to Kelly et al. A Comparison of Brain-State Representations of Binary Neuroimaging Connectivity Data. Comment on “Samantaray et al. Unique Brain Network Identification Number for Parkinson’s and Healthy Individuals Using Structural MRI. *Brain Sci.* 2023, *13*, 1297”

**DOI:** 10.3390/brainsci14050424

**Published:** 2024-04-25

**Authors:** Tanmayee Samantaray, Manish Anand, Jitender Saini, Cota Navin Gupta

**Affiliations:** 1 Neural Engineering Lab, Department of Biosciences and Bioengineering, Indian Institute of Technology Guwahati, Guwahati 781039, India; tanma176106113@iitg.ac.in (T.S.); manish_anand@iitg.ac.in (M.A.); 2 Department of Neuroimaging and Interventional Radiology, National Institute of Mental Health and Neurosciences, Bengaluru 560029, India; jsaini76@gmail.com

**Keywords:** structural MRI, UBNIN, Parkinson’s disease

## Abstract

The purpose of this reply is to address the comments given by Kelly et al. on our original paper “Unique Brain Network Identification Number for Parkinson’s and Healthy Individuals using Structural MRI”. We agree to the inadvertent rounding pitfall in our original paper due to the non-inclusion of symbolic math toolbox (MATLAB). We now provide the actual ranges (with decimal values) of the UBNIN values of healthy individuals and those with Parkinson’s disease and further observations. Upon further introspection, we propose another variant, called Modified-UBNIN (UBNIN-M_T,MN_) which is highly weighted on the node with the highest network degree (i.e., connections). The italicized sentences within inverted commas are statements from Kelly et al.’s comment paper.

We appreciate Josephine N. Kelly et al. for their interest and for taking time to investigate our work [1] in detail. We are glad our work [1] sparked a scholarly conversation. We hereby reply to all of their remarks [2], which we are sure will help clarify all their points and improve our paper’s level of detail.

We proposed a novel algorithm, namely, Unique Brain Network Identification Number (UBNIN) in our study [1] for representing individual brain networks using the regional grey matter volume (rGMV in cm^3^) from structural magnetic resonance imaging (sMRI) scans of the human brain.

We [1] used sMRI scans of each individual brain to construct respective adjacency matrix comprising binary data, where 1 represents the presence and 0 represents the absence of connections between nodes (brain regions). Further, we appreciate that Kelly et al. have proposed variants of UBNIN, naming them UBNIN-C (complete UBNIN), UBNIN-R (Rounded UBNIN), and UBNIN-T (Truncated UBNIN). However, we would also like to emphasize that UBNIN-T, as understood by Kelly et al. from our abstract, is not a truncated version of UBNIN. Rather, it is the complete UBNIN value obtained. The UBNIN value (in our original paper) lacks the fractional part due to rounding off in the intermediate steps during computation, which we have now recovered using the symbolic math toolbox in MATLAB. We have not purposely truncated any UBNIN value in the algorithm to make it manageable. The UBNIN range for the HC and PD subjects are now added in the appendix of this paper. This also explains the following query that was raised: “*Interestingly, the UBNINs shown… retrieved by decoding those UBNINs*” and “*It is not clear exactly what the authors… to understand its properties*”. Additionally, we would like to mention an error in Figure 1 of Kelly et al., displaying ‘1′ in row 9, column 8 and in row 10, column 8. Neither Figure 1 nor 2 of our original article shows a connection between the eighth and ninth node or the eighth and tenth node.

We appreciate that Kelly et al. have provided further insights into the UBNIN by providing a comparison between the UBNIN and other methods (binary, base 10, and hexadecimal representations). However, we would like to clarify that information on the number of nodes is required for decoding in a hexadecimal way of representation, unlike what is mentioned in Table 2 of the comment paper by Kelly et al. This is because the number of digits in this representation does not imply the number of nodes. Moreover, our UBNIN method indicates the number of nodes in UBNIN_T_, where T denotes the number of nodes in the network or columns in the adjacency matrix. Hence, extra information is already available in UBNIN_T_, as the subscript ‘T’ determines the correct power of 2. Additionally, as mentioned by Kelly et al., we also obtained the same UBNIN value of 321.005979848894639872014522552490234375. However, this number was too long for representation, hence we showed only up to 15 digits. In response to “*Toward this end… base 10 representations of that column*”, it is worth noting that we did not reorder in the way explained in the above lines to preserve the original order of nodes, as they denote specific brain regions. The sequence of nodes for each subject is hence kept identical to those of every other subject and is the same as that obtained using a standard parcellation atlas (LPBA40, [3]). Thereby, with reconstruction, no additional data would be required to map the brain regions.

We also propose that in UBNIN-M_T,MN_, the MN (maximally occurring principal node) will play a major role and can be selected from the data or literature. It could be the most significant brain region differing between health and disease (e.g., substantia nigra for Parkinson’s disease and superior temporal gyrus for schizophrenia).

The algorithm for generating the Modified Unique Brain Network Identification Number (UBNIN-M_T,MN_) builds upon the original UBNIN algorithm with small modifications. This new algorithm uses the binary adjacency matrix (AM), illustrated in Figure 1, with 12 nodes. We evaluate the node degree, which is the number of connections (ones) in each column (node) of the AM (Step 1, Figure 1). The node with the highest degree (highlighted in pink, Step 2, Figure 1), is denoted as the principal node. This principal node for every healthy control (HC) was determined and the node with the maximum occurrence across the entire HC group is designated as the maximally occurring principal node (Figure 2). The same procedure was followed for the patients with Parkinson’s disease as well.

Further, the lower triangular matrix is zeroed out, as the matrix is symmetric along the diagonal and the network is undirected (region shaded in green, Step 3, Figure 2). Now, each of the columns, from bottom to top, is assumed to be a singular binary number (Step 4, Figure 2). The values in the binarized column of the AM are then converted into their respective decimal equivalents (Step 5, Figure 2). Thus, the values (*DEC*) obtained are 0, 1, 3, 6, 13, 20, 60, 67, 1, 321, 503, and 996. The maximally occurring principal node has a *DEC* value of 3. The Modified-*UBNIN* is calculated in a similar way to the original *UBNIN*, except that the *DEC* value of the maximally occurring principal node is skipped over in the sequence (Equation (1)). The *DEC* of the maximally occurring principal node constitutes the integer part and the remaining *DEC* values were turned into a fractional value using Equation (1), where *T* is the total number of nodes in the network, *MN* is the maximally occurring principal node of the network, and *i* is the node/column number of the network/binary matrix.

(1) $${UBNIN-M}_{T=12,MN=3}=\frac{1}{2^{i=12}}\times \left(\frac{1}{2^{i=11}}\times \left|\frac{1}{2^{i=10}}\times \left[\frac{1}{2^{i=9}}\times \left\lceil \frac{1}{2^{i=8}}\times ⟦\frac{1}{2^{i=7}}\times \left\{\frac{1}{2^{i=6}}\times \left\|\frac{1}{2^{i=5}}\times \left(\frac{1}{2^{i=4}}\times \langle \frac{1}{2^{i=2}}\times \left\lfloor \frac{1}{2^{i=1}}\times DEC\left(1\right)+\;DEC(2)\right\rfloor +DEC(4)\rangle +DEC(5)\right)+DEC(6)\right\|+DEC(7)\right\}+DEC(8)⟧+DEC(9)\right\rceil +DEC(10)\right]+DEC(11)\right|+\;DEC(12)\right)+DEC\left(3\right)$$

Upon substituting the values in Equation (1) with the respective nodes and decimal values obtained in Step 5 of Figure 2, we obtain


$$\begin{array}{l} UBNIN-M_{12,3}=\frac{1}{2^{12}}\times (\frac{1}{2^{11}}\times [\frac{1}{2^{10}}\times [\frac{1}{2^{9}}\times \lceil \frac{1}{2^{8}}\times ⟦\frac{1}{2^{7}}\times \{\frac{1}{2^{6}}\times ∥\frac{1}{2^{5}}\times (\frac{1}{2^{4}}\times \langle \frac{1}{2^{2}}\times \lfloor \frac{1}{2^{1}}\times 0+1\rfloor +6\rangle +13)+20∥+\\ 60\}+67⟧+1\rceil +321]+503]+996)+3 \end{array}$$

The resultant Modified-*UBNIN* (*UBNIN-M_T,MN_*) for the above 12 nodes (*UBNIN-M*_12,3_) is 3.243284136525345895659473878513967548542495933361351490020751953125. The expression $\frac{1}{2^{i}}$ starts with i=1, and the equivalent *DEC* value starts with the first node of the matrix. Thus, the node with the highest degree and maximum occurrence in a cohort is highly weighted in the final *UBNIN-M_T,MN_* value. The *UBNIN-M_T,MN_* range for HC and PD is provided in the Appendix A.

In response to “*Finally, the word Unique… not guaranteed to differ*”, we would like to convey that the UBNIN algorithm did seem to provide a unique value for every individual considered in the healthy control (HC, *n* = 70) and Parkinson’s disease (PD, *n* = 179) groups. We have also employed the UBNIN algorithm and found distinct UBNIN values from 100,000 iterations of randomly assigned (i.e., not of a specific sparsity) 10 × 10 binary matrices. It was hence termed the ‘UNIQUE’ Brain Network Identification Number. This is also mentioned in our original paper (Section 4.1) [1]. Using the symbolic math toolbox in MATLAB and the Modified-UBNIN, seven HC and two PD individuals seemed to have similar values, which could be due to the similar connectomes in the brain morphology. The reason behind these similarities needs further investigation.

Considering Takao et al.’s work [4], Kelley et al. seem to suggest “*Even MRI scans for the same person… Not for each person*”. Takao et al. [4] performed a longitudinal study to evaluate the inter-scanner variability and effect of MRI scanner drift. However, the uniqueness defined in our paper [1] was obtained in a cross-sectional study using T1-weighted MRI scans from a single site and single scanner. We do acknowledge that there could exist subtle differences in the UBNIN for the same person (say Mr. X) due to scanner drift when two scans are taken at a different time. We also speculate that the scans of two people (Mr. X and Mr. Y) and thereby the UBNIN could vary considerably due to the inherent grey matter differences in the human brain. However, the preceding two statements need more investigation and we invite Kelly et al. to investigate and work with us on this idea if they have access to such a dataset. In this context, it might be relevant to mention that Meda et al. found little effect of the sites (1.5 T scanner of same model at four sites) on grey matter differences in schizophrenia patients [5] using cross-sectional MRI data. Stonnington et al. [6] used cross-sectional data (six 1.5 T scanners of the same vendor at one site) to investigate the grey matter differences in Alzheimer’s disease and found substantially less scanner differences than group differences. We would also like to speculate that a change in the UBNIN value might have a diagnostic viewpoint, as mentioned in Section 4.1 [1], as was observed with the UBNIN variation between the HC and PD individuals.

The size of brain regions in the LPBA40 atlas vary significantly. This may affect the connection strength in brain networks due to regional volume differences in individuals. This motivates further investigation using approaches such as uniform seed size [7]. We presume that seed-based network analysis using the Modified-UBNIN holds promise for brain printing and biomarker discovery.

We once again appreciate the in-depth analysis of the UBNIN algorithm by Kelly et al.

## Figures and Tables

**Figure 1:**
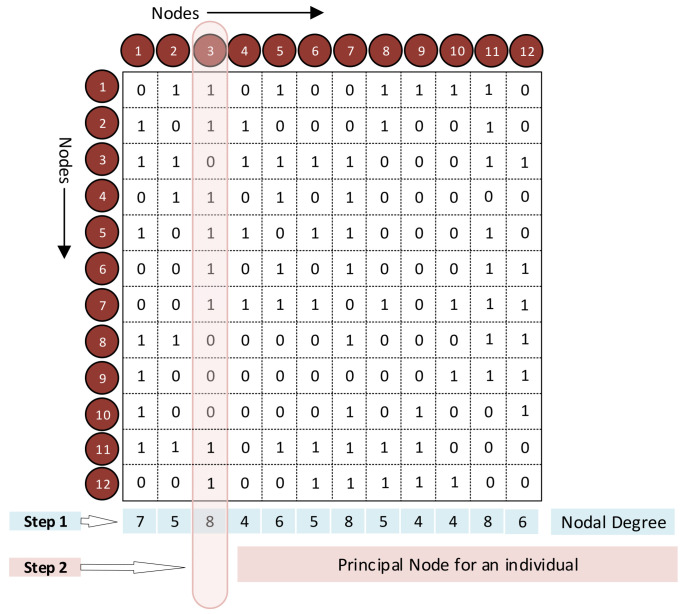
Modified-UBNIN (UBNIN-M_T,MN_). Estimation of principal node.

**Figure 2:**
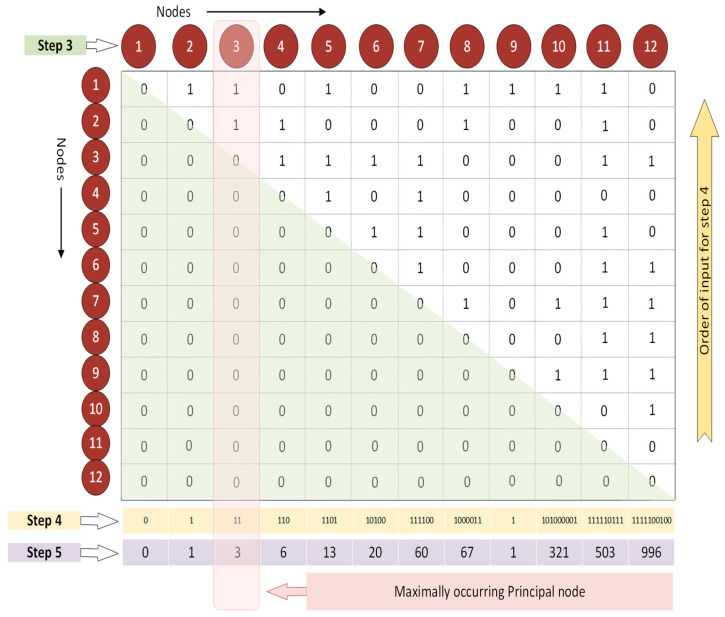
UBNIN-M_T,MN._ Estimation of maximally occurring principal node and computation of Modified-UBNIN value.

## Data Availability

The imaging and clinical data were collected from the National Institute of Mental Health and Neurosciences and may be provided upon approval as per Institute norms. The code developed for this work is available at our GitHub link below: https://github.com/NeuralLabIITGuwahati/Modified_UBNIN (accessed on 23 April 2024).

## Appendix A

Minimum UBNIN_T_ values from PD patients:
  15360.000000000000000054188109290694681711540712349210283763615674543563352373044540314108307985417491542032568424089945443628662866518209823592963211798055392418162866440660500025346014631897935368865621231487209789819639232259485788718735790620537439107397323320269853958708899302064421433379070058614486583996106145227420852012700010002100292513335806451716586467312577373430032405405709877243052223172001463471249460393515309974607551173850761995674676909913060407007996421542094139163344766893703494382069663496081822992974186848686111809685133602367325067951311474572261149742768387215023684837157623923840284894818543966267758303940579136168736092793987024797736026725595802072465005209276994730676643041424141693314163997495186791647437546106082840102075377487848908286608858703783611446419176636876164494608780440495921331760578235732326495031020115232033649544357473581772705370553134390966429074562091010032732642368795718691604905058325640575157871358356089223045694972474627864622646876163973127544879815996921352647417315065902620930914513062406797466111827341205712147583680713979575850121725871100442120550809232491103670867722231603453735922521439630608456247536537216746957068341100316426200325196956050415686657728150591920606024279780254364662481608129761044742783175233251003138002420489476381931661623221427457494579818808502891866628524240043355535524610942609880980657108019353781969241618526931109274951209110588857752495027704509045207714734715409576892852783203125
Maximum UBNIN_T_ values from PD patients:
  17768936615460608.000000000000000054161005916593163343009832617230468113674246047280012246408829546387199112184955047207358311040032881652515738147739248264000913181044790234818623649945566330112305715469600159347660290377162354170751901752942061797522479615220838398299435975356903179690234644466399566464120043976482947470747761172799373795039801873375790989077193154066812849603900383032074286352900651700895916477248245782744775467062163262778463621065725988180616115285767677223412989059859677550693314592575724303710517810869697622782314161414348442804577977709795772373661272709254950516276552569103975328461295681242171432770079334451131422472592590903205707690984591354826349037318681120326542978659556163500556206116169888232626935199684783276708976623132850953983103579499825062327181147571115635842163292709653678494294780894132133914309925621792983219752418811187857672760798528930506700950042156077652276203436633810023860687087496878190753558928052830214496431008682800939248849110456633075352730972484902939719126071222422985387645315496839366686460619628411359764384007002617434368899768341319239553971313918403585877861841611518905299872537333784539187626440532290979302129799532053317636867047618811222111763711338955969601472682243801649865083859332793793442046159041328689969082192935751992757608234116541780621956554991878126742133041030134315361160817588799793546796130310603111565449829496126136440279267066968033352320764501742699243845742540504062390027684159576892852783203125
Minimum UBNIN_T_ values from HC subjects:
  12288.000000000000000054106795756270099824708156665866678597694453410061297132624468683434161970758629330185394426308045619802420004248780802617018084752258162368433073792884229621380081496543486157186728682507166150568632830866725370597355265509904749165711576438772020481799046492330113804477286514038126385698520210733348984936184815040083389673855326389015731931104410351369328894800511297409077876533450266016123245178647509464388162135194497190675383710986941299803441486640366201632425993916806853418055946306080584093002338298079971059566593125943777436993128446445737433699791444185162906106960140354047162921428296783597723492048459322004385870464606519289804044340284791109698243417771728595570889590502117662236078876410334679382535179925940813393002493000738210700402412238114412949300577779516563664592844549752948794491973298518914959150589150296327405663950565338386879568039799006767032761461373877501834403693081989412719520292905170721215178211744528858078484546412766455356135229784300560789002694130277550147970006179607373409563528598723800486895371628480529919868767750051368641576616974223375285256390486648379391778371720983853140457178140621259492744976815135775299924882093025707974917588313655995565749058790499302783581027523477051608226980300163344906265800997559890240179937417449242215015269855846254530571454746870881691588811013837177508838039476773136522454601722863201204365684245744676993071826385923573864451395672159339511608777684159576892852783203125
Maximum UBNIN_T_ values from HC subjects:
  17733751438064640.000000000000000054120348257576774496878925860742690765029916703066340407452168187419647341853151022767251464361357852396515113619741971631739794261206800703097794654538420886038233144144286873629854620833748683176513559142616774721867226550682213477792136035263694200097826678126465584507670723172624556991649365343609563839284715090638240407876142956237493780843015828676310143203703414335704100394239697698511895748326074473836296855050382508788033335734791089438321692458621634333649081591932277475205230436096070859818209011886874063829777814864422668774840504207434071549548434115607434283204876578563529885495216805998967980040828964324526181816720890552574459273339635394349653383895858410799147332543673426103202917476430439488511646880868440858965058957946868089285706907922003110407520811710497690370530695471826049662278424543823736533069781739853650264740713108383169738217576708839978370875687864788661310146031818995882427901525058541074884126813546100133209531239087433555966209647711464439339681368621344570557758221028631908679037726417030238316439229699365945970521195595419954011898173934670370722816273015032596918763552026748467672049662756200636645287155801001498491899469597186790061423863637172843987288280388884948994720576010921443955946036876236310381801406949849713390584554364916004622835090941625601112386421032464971225354016229270333487922002636106815454953416655833555156060961350522258906620627478668493799273016275908076977430027909576892852783203125
